# 

**Published:** 1887-03

**Authors:** 


### CONTENTS.

COMMUNICATIONS.

Tumors of the Mouth........ 109
Is Life Force Matter?........ 112
Cohesive Foil........ 117
The Teeth as Dermal Apendages........ 119
Syphilis—Signs, Symptoms, and Treatment........ 125
Historical Sketch of the Mississippi Valley Association of Dental Surgeons........ 131
Crown and Bridge Work........ 141
CORRESPONDENCE........ 144
INTERNATIONAL DENTAL CONGRESS........ 146
Ninth International Dental Congress........ 148
International Medical Congress—Trans-Atlantic Rates........ 152
SALMAGUNDI........ 153
Proceedings of Societies........ 157

EDITORIAL.

Bibliographical........ 158
A New Motor for the Dental Profession........ 160

